# Intersection of neighborhood dynamics and socioeconomic status in small-area walkability: the Heart Healthy Hoods project

**DOI:** 10.1186/s12942-017-0095-7

**Published:** 2017-06-06

**Authors:** Pedro Gullón, Usama Bilal, Alba Cebrecos, Hannah M. Badland, Iñaki Galán, Manuel Franco

**Affiliations:** 1 Social and Cardiovascular Epidemiology Research Group, School of Medicine, University of Alcalá, Alcalá de Henares, Madrid 28871 Spain; 2 Escuela Nacional de Sanidad, Instituto de Salud Carlos III, Madrid, 28029 Spain; 3 Department of Epidemiology, Johns Hopkins Bloomberg School of Public Health, Baltimore, 21205 MD USA; 4 Department of Geology, Geography and Environmental Sciences, University of Alcalá, Alcalá de Henares, Madrid, 28871 Spain; 5 Center for Urban Research, RMIT University, Melbourne, 3000 VIC Australia; 6 Centro Nacional de Epidemiología, Instituto de Salud Carlos III, Madrid, 28029 Spain

**Keywords:** Physical activity, Neighborhood/pace, Urbanisation, GIS

## Abstract

**Background:**

Previous studies found a complex relationship between area-level socioeconomic status (SES) and walkability. These studies did not include neighborhood dynamics. Our aim was to study the association between area-level SES and walkability in the city of Madrid (Spain) evaluating the potential effect modification of neighborhood dynamics.

**Methods:**

All census sections of the city of Madrid (n = 2415) were included. Area-level SES was measured using a composite index of 7 indicators in 4 domains (education, wealth, occupation and living conditions). Two neighborhood dynamics factors were computed: gentrification, proxied by change in education levels in the previous 10 years, and neighborhood age, proxied by median year of construction of housing units in the area. Walkability was measured using a composite index of 4 indicators (Residential Density, Population Density, Retail Destinations and Street Connectivity). We modeled the association using linear mixed models with random intercepts.

**Results:**

Area-level SES and walkability were inversely and significantly associated. Areas with lower SES showed the highest walkability. This pattern did not hold for areas with an increase in education level, where the association was flat (no decrease in walkability with higher SES). Moreover, the association was attenuated in newly built areas: the association was stronger in areas built before 1975, weaker in areas built between 1975 and 1990 and flat in areas built from 1990 on.

**Conclusion:**

Areas with higher neighborhood socioeconomic status had lower walkability in Madrid. This disadvantage in walkability was not present in recently built or gentrified areas.

**Electronic supplementary material:**

The online version of this article (doi:10.1186/s12942-017-0095-7) contains supplementary material, which is available to authorized users.

## Background

A quarter of the population in Europe is estimated to be physically inactive [1]. Reducing physical inactivity is one of the key targets to control non-communicable diseases [2] as it is estimated to be responsible for 6–10% of the burden of major non-communicable diseases worldwide [3]. Cities, due to the possibility of population approaches [4, 5], represent an opportunity for public health interventions on physical inactivity [6, 7].

Walkable neighborhoods (dense, compact, with availability of walking destinations) are associated with improved walking behaviors [8–10]. In addition, physical inactivity follows a social gradient, with more disadvantaged populations having a higher prevalence of physical inactivity [11–13]. Thus, the interaction between urban form (defined as physical form of the city [14]) and social disadvantage could provide insights on how socio-spatial inequalities in physical activity are shaped [15]. Previous evidence suggests that the relationship between Neighborhood Socioeconomic Status (SES) and neighborhood walkability may be complex [16]. In particular, previous research has found that lower SES neighborhoods are more walkable (measured using objective measures) [16–19], while on the other hand residents of more deprived neighborhoods report worst aesthetics and safety of their neighborhoods [20, 21], which may also be important contributors to walking behaviors. However, most of the studies looking at the relationship between social and urban form have been conducted mainly in the US and Australia, where the shape of urban environments [22] and socioeconomic segregation processes [23] differs widely from European cities.

Moreover, neighborhoods and cities are not static entities, they are dynamic in its form and composition [24]. Urban form changes at a slower pace than social composition, as citizens might move following a social gradient in response to changes in the housing prices market [25]. At the same time, urban form tends to change at a slow pace, given its constrains in some parts of the city (e.g. inner old city). In order to understand the socio-spatial inequalities in walkability there is a need to incorporate variables that can help to understand the dynamics and the history of the city. In our study, we try to encompass this challenge by incorporating variables of change in social composition and the age of the neighborhood.

The Heart Healthy Hoods project (HHH) aims to study how urban environment relates to cardiovascular health of Madrid’s residents [6, 26, 27]. Within this project, some measures of physical activity environment have been tested [28]. Taking all the above into consideration, our aim was to evaluate the association between small area-level socioeconomic status and walkability in the city of Madrid (Spain) and to evaluate the potential effect modification by indicators of neighborhood dynamics (gentrification and neighborhood age).

## Methods

### Study setting

We conducted our study in the City of Madrid, Spain. In 2014, Madrid was divided into 21 districts that housed 128 neighborhoods, that were further divided into 2415 census sections [29]. The Census Section was the unit for all the analysis as this is the smallest area for which census and other relevant data were available. Census Sections had resident populations of between 1000 and 1500 people. Madrid’s socio-spatial configuration is one of the most segregated in Europe [30]. As most of European cities, it has a historic city center, and neighborhoods separated from it by an orbital motorway [31]. Since the 60s, it has experienced a huge economic and population growth due to the industrialization of some parts of the region and the migration from rural areas. Higher social class tend to accumulate in the northern part of the city [30].

### Area socioeconomic status

The main exposure of this study was a composite SES index made of 7 indicators. These were: (1) Low education (defined as % people above 25 years of age with primary studies or below), (2) High education (defined as % people above 25 years of age with university education or above), (3) Part-time employment (% workers in part-time jobs), (4) Temporary employment (% workers in temporary jobs), (5) Manual occupational class (% workers in manual or unqualified jobs), (6) Average housing prices (per sq. m), and (7) Unemployment rate. These indicators were selected based in the 4 domains present in the Spanish Commission to Reduce Health Inequalities [32] (education, wealth, occupation and living conditions). Using this framework, the SOPHIE project have investigated the effect of structural policies on health inequalities [33]. Indicator data were obtained from the Padrón (a continuous and universal census collected for administrative purposes), the social security and employment services registries and the IDEALISTA report (a report from a large real estate corporation in Spain). All data were available for the year 2014. Table 1 (and Additional file 1) contains more details on the operationalization of indicators.

**Table 1:** Area Socioeconomic status, Walkability and neighborhood dynamics indicators

Construct	Domain	Indicator	Operationalization	Source	Level
SES	Education	Low Education	Residents with mandatory studies or below/all residents aged 25 or above	Padron	Census section
		High Education	Residents with university education or above/all residents aged 25 or above	Padron	Census section
	Occupation	Part time Jobs	Workers in part-time jobs/all workers	Social security	Neighborhood
		Temporal Jobs	Workers in temporal jobs/all workers		
		Manual Occupation Class	Workers in manual or unskilled occupations/all workers		
	Wealth	Housing Prices	Average sale price of housing per m^2^	Idealista report	Census section
	Living Conditions	Unemployment Rate	Residents registered as unemployed/all residents aged 16–64	Employment service	Neighborhood
Walkability	Density	Residential Density	Occupied Dwellings/km^2^	Housing census	Census section
	Density	Population Density	Residents/km^2^	Padron	Census section
	Destinations	Retail Destinations	Retail and Service Destinations/km^2^	Retail spaces census	Census section
	Street Structure	Street Connectivity	Kernel Density in 3 mx3m pixels of the density of street intersections	CARTOCIUDAD	Census section
Neighborhood dynamics	Gentrification	Increase in Education level	Rank difference in high education from 2005 to 2014 (>p95)	Padron	Census section
	Neighborhood age	Year of construction	Median year of construction (categorized)	Catastro	Census section

To create the SES index, we constructed a weighted index from the variables described above. For this we centered (to the mean) and scaled (by the standard deviation) all selected variables. We then weighted the four domains equally (0.25 per domain) and weighted all variables within each domain equally (e.g.: overall, each education variable has a weight of 0.25 × 0.5 = 0.125). We then averaged all standardized variables to obtain the SES Index. We compared this index to a score obtained using the principal component of a Principal Component Analysis and found a Pearson correlation of 0.997 between them.

### Neighborhood dynamics

For neighborhood dynamics, we selected 2 indicators: gentrification and neighborhood age. An indicator for gentrification was obtained by ranking all census sections in 2005 and in 2014 in terms of % residents with high education (university education or above) and computing the change in rank from 2005 to 2014, where we defined a gentrified neighborhood as those in the top 95% percentile of rank change. Neighborhood age was proxied by the median year of construction of all housing units in the census section, obtained from the Cadastre (*Catastro*, a universal tax registry of all housing units). We created three categories: up to 1985, from 1985 to 1997, from 1997 onwards. These categories were created based on the time of creation of the land-use planning regulations of the city [34]. Table 1 (and Additional file 1) contains more details on the operationalization of indicators.

### Walkability

A walkability index for the 2415 census sections was created, reflecting known barriers and promoters to walking behaviors [9]. The core components of walkability indexes are the presence of places to walk to, a street network that facilitates such walking and enough density to guarantee that destinations are not too far apart [9]. Based on these, many previous measures of walkability have been developed [35]. Here, we used an index based on work by Creatore et al. [36] with modifications based on European recommendations [37]. The following indicators were used: Residential Density (occupied dwellings/km^2^), Population Density (Residents/km^2^), Retail Destinations (Retail and services destinations/km^2^) and Street Connectivity (Kernel Density in 3 m × 3 m pixels of the density of street intersections). Data were obtained from the Housing part of the Spanish Census (that includes data on occupied dwellings), the Padrón (sociodemographic data), the Retail Spaces Census (curated by the local government for licensing purposes, that includes data on economic activities of all occupied commercial spaces) and CARTOCIUDAD (the National Mapping Agency initiative that collects and makes available official geo-referenced urban data, including street structure and administrative boundaries in shapefile format). All data were available for 2014 except for the Spanish Census, available for 2011. Table 1 (and Additional file 1) contains more details on the operationalization of indicators. To create the walkability index, we followed the same procedure as for the exposure (see above), and weighted the four indicators equally.

### Statistical analysis

The objective of this analysis was to study the association between area-level socioeconomic status and area-level walkability, and how neighborhood dynamics influence these associations. We conducted exploratory and descriptive analysis of the exposure and the outcome variables, by tertile of neighborhood SES (Additional file 2: Table S2). We also plotted the distribution of SES and walkability indexes and examined their association using a non-parametric *lowess* [38] estimator, to provide an idea of the best operationalization of the neighborhood SES indicator.

To study the association between neighborhood SES and walkability we used linear mixed models with the walkability index as the dependent variable. These models included a random intercept for neighborhood (as census sections are nested into neighborhoods). We assessed whether a third level (for district) was needed by adding a random intercept for district and performing a likelihood ratio test of the nested models. Afterwards, we further included the SES Index operationalized as deciles, with the sixth decile as the reference. Based on the exploratory analysis above, we also conducted an analysis where we modeled the association using restricted cubic splines with 5 knots placed in the percentiles recommended by Harrell [39]. The number of knots was decided after testing 3–6 knots models and selecting the best fitting model based on Akaike Information Criterion (AIC).

We tested for effect modification by neighborhood dynamics indicators by adding an interaction term between the gentrification or neighborhood age indicator(s) and each restricted cubic spline. We checked for the significance of this interaction by conducting a likelihood ratio test in nested models with and without the interaction. All analyses were conducted using Stata SE version 14.1 (StataCorp., College Station, TX, USA).

## Results

### Spatial distribution of SES and walkability

**Fig. 1:**
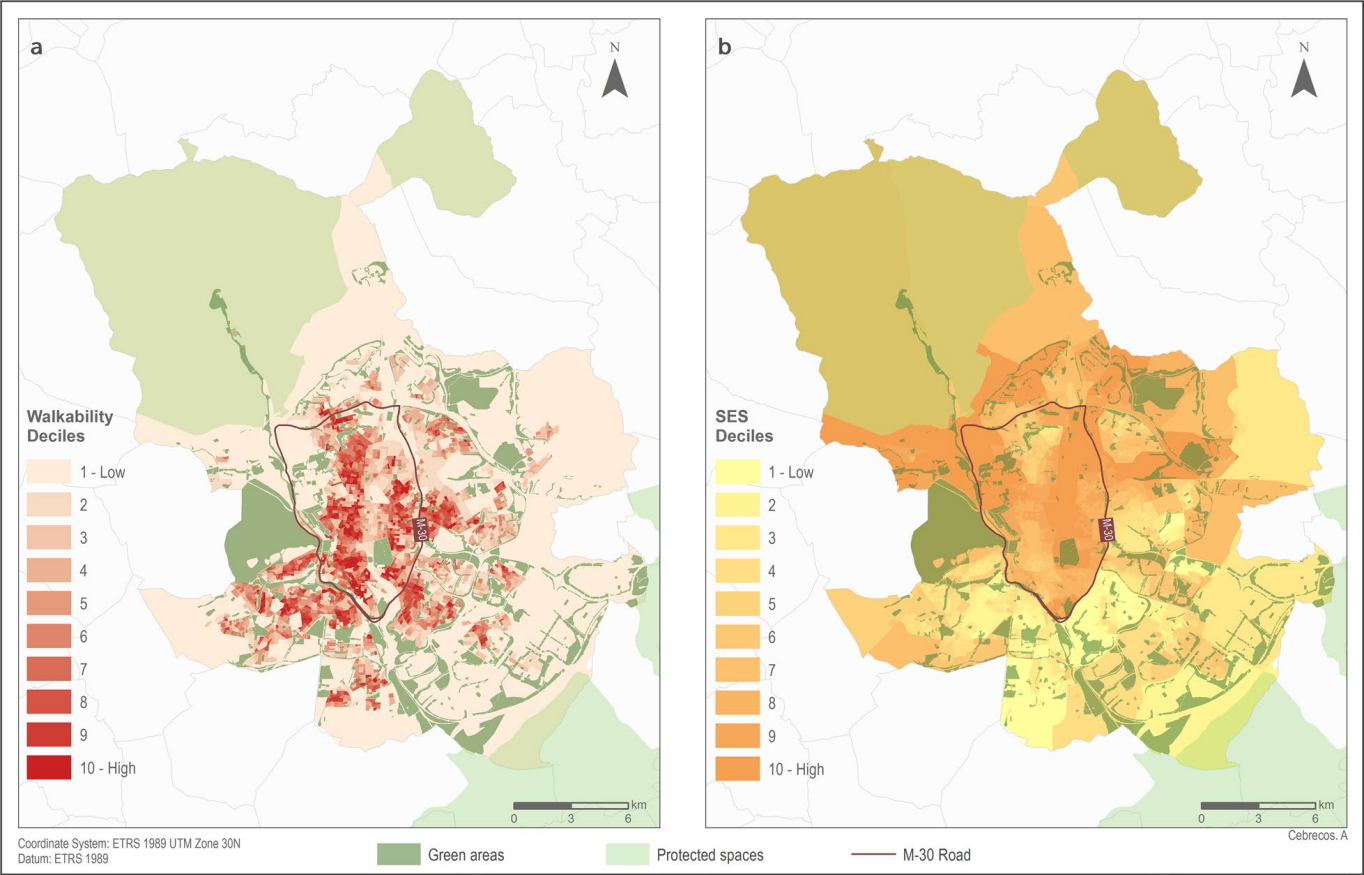
Spatial distribution of Walkability Index (**a**) and Socio-Economic Status Index (**b**) by deciles in the census section (N = 2415) of the city of Madrid

The spatial distribution of SES and walkability indexes is shown in Fig. 1. Walkability was higher in the downtown area of Madrid (inside the M-30 orbital motorway of Madrid). There are also some pockets of high walkability in the areas adjacent to the M-30 orbital motorway, especially in the Southeastern and Southwestern parts of the city. Socioeconomic status followed a major North–South decreasing gradient (higher SES in the Northern areas of the city), while the Southern peripheral neighborhoods of the city had a lower SES.

### Association between SES and walkability

Table 2 shows the main results of the study, obtained from a three-level mixed effects model. All SES Index deciles showed statistical significant differences compared to the reference group (sixth decile). Lower SES census sections had the highest walkability: there was an increase of 2.19 SD (CI 95% 1.36; 3.01 p < 0.001), 2.87 SD (CI 95% 2.19; 3.54 p < 0.001) and 2.02 SD (CI 95% 1.46; 2.59 p < 0.001) of walkability in the first, second and third decile of SES respect to the reference group. Fourth and fifth SES-deciles also ranked higher in walkability than the reference. Higher SES deciles had lower walkability: there was a decrease in walkability of 2.93 SD (CI 95% −3.60; −2.26 p < 0.001) and 3.86 SD (CI 95% −4.61; −3.12 p < 0.001) for the ninth and tenth SES-deciles.

**Table 2:** Results from multilevel regression analysis between area SES Index and walkability Index (N = 2415 census sections)

SES Index decile	β	95% CI	p value
1	2.19	1.36; 3.01	<0.001
2	2.87	2.19; 3.54	<0.001
3	2.02	1.46; 2.59	<0.001
4	1.26	0.73; 1.80	<0.001
5	0.53	0.06; 0.99	0.027
6		Ref	
7	−1.65	−2.17; −1.13	<0.001
8	−2.23	−2.85; −1.61	<0.001
9	−2.93	−3.60; −2.26	<0.001
10	−3.86	−4.61; −3.12	<0.001

**Fig. 2:**
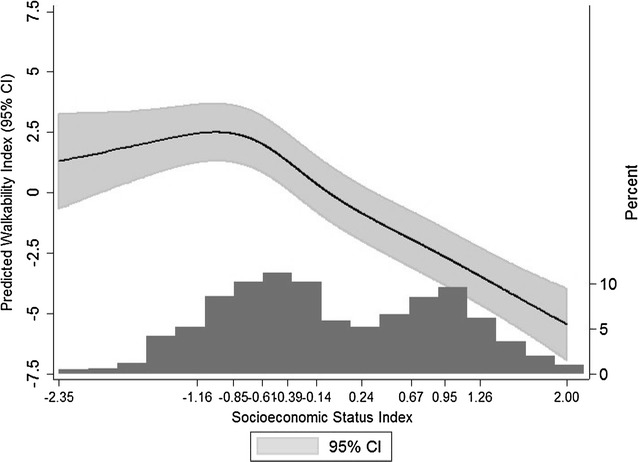
Restricted cubic splines with 5 knots placed in the percentiles recommended by Harrell [39] showing the relationship between SES and walkability indexes in all Madrid census sections (N = 2415). X axis represents the min, max and 10 deciles of the SES Index, and Y axis represents the predicted Walkability Index. The* line* represents the predicted walkability through SES level and its 95% CI.* Histogram* represents the SES Index distribution of the 2415 census sections

Figure 2 shows the results of the model using restricted cubic splines with 5 knots. This figure shows a dose–response association for most of the SES distribution, with the only exception of a slight change in the association in the lowest tail of SES, where walkability decreases as SES decreases.

### Interaction by neighborhood dynamics

**Fig. 3:**
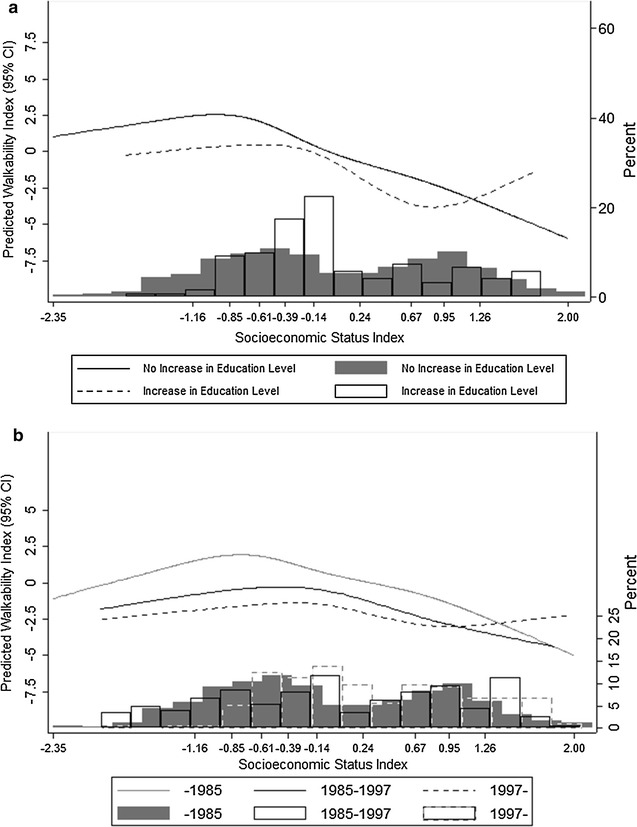
Interaction effect by indicators of neighborhood dynamics (**a** gentrification,** b** neighborhood age) using restricted cubic splines with 5 knots placed in the percentiles recommended by Harrell [39]

Figure 3 shows the results of the interaction with neighborhood dynamics. Panel A shows the analysis by change in education level (gentrification), where we found a significant interaction between SES and change in education level (p < 0.001): while areas with stable or decreasing education level showed the same overall pattern (decrease in walkability with increasing SES), areas that increased its education level had a flat pattern with no association between neighborhood SES and walkability. Panel B show the analysis by neighborhood age, where we also found a significant interaction (p < 0.001). The association was similar in shape for areas built before 1985 and between 1985 and 1997 (albeit these second group showed an overall decrease in walkability regardless of SES). Areas built after 1997 showed a flat association, with no decrease in walkability in areas with higher SES.

## Discussion

Our results indicate that census sections with higher socioeconomic status had a less walkable-urban form, as defined by our Walkability Index. This association followed, for the most part, a dose–response linear pattern. Moreover, we found that this association is heterogeneous, as there are significant interactions by a marker of gentrification and neighborhood age.

The negative association between area SES and walkability has been found in other studies [16–19, 21]. For example, Carpenter et al. [19] found a positive relationship between street connectivity and neighborhood poverty. King et al. [16] found that disadvantaged neighborhoods in terms of median income were more walkable (shorter block length, greater street node density, more developed land use, and higher density of street segments); on the other hand, they also found that more educated neighborhoods were also more walkable [16]. In our study, we created a composite Index, which allow us to better measure SES by using information from several indicators and therefore reduce the degree of measurement error. Other studies found that residents of more deprived neighborhoods report worst aesthetics and safety of their neighborhoods [20, 21]. Mixed-methods between quantitative and qualitative methods [40] could represent an alternative in order to understand the different associations between objective and subjective walkability measures.

We found an interaction with our marker of gentrification (top 95% percentile of rank change in high-education level in the last 10 years). Non-gentrified areas showed an inverse association between SES and walkability, while gentrified areas show a flat association between SES and walkability. As opposed to non-gentrified areas, where a “disadvantage” [16] in walkability was evident for higher-SES census sections, this phenomenon was not present in gentrified areas where higher-SES census sections had a similar walkability as lower SES ones. One potential explanation for this phenomenon is an increasing popularity of walkable neighborhoods (reflected by the increase of housing prices), where lower-SES residents are not able to continue living due to a decrease in affordable housing, causing a replacement of lower-SES residents for higher SES residents [25]. These gentrification and urban renewal processes have launched popular movements and social mobilization against them [41].

We also found an interaction by neighborhood age (assessed by our indicator of median year of building construction). The newest areas (those with its median year of construction after 1997) had a flat curve compared with the older neighborhoods (built before 1997). Independent of SES, historic and old neighborhoods tend to have a greater walkability due mostly to a denser street network [42]. Similarly to our gentrification analysis, there is a lack of a walkability disadvantage in higher SES areas built from 1997 onwards. Conversely, there is a lack of an “advantage’ in walkability in lower SES areas, probably reflecting the newer developments of lower SES housing in the periphery of Madrid, with a less dense street network and lower availability of destinations. Recent research has shown an initiation of sprawling patterns in Mediterranean cities the last decades [43].

This study has several strengths. First, as far as we know, this is the first study to explore the walkability-SES association in an Southern European setting, characterized by its overall higher density [22], and to look at what is the effect that social and urban form dynamics have on it. We have also built a strong SES and walkability measures using GIS and an integrated composite index, which allow us to better measure SES by using information from several indicators and therefore reduce the degree of measurement error. Our walkability Index has been adapted for one used in Canada [36], but the changes followed some adaptations that could be needed for European context, using different measures for connectivity and land-use [37]. Most of the walkability literature has been conducted in the US, Canada and Australia or New Zealand, in cities with a much shorter lifespan than European cities [9]. We believe that conducting this type of research in European cities with a longer historical trajectory, a presence of different urban form structures (like historical mixed-use downtown areas) provides a different mechanistic insight into the determinants of walkability and can help inform policies in European cities in a more appropriate way.

This study has some limitations. We only measure the association of area-SES with walkability, a very specific set of features that promote walking behaviors, and may be missing another physical activity environment measures which could be important for public health, such as perception of crime and safety which are important determinants specially in low SES areas [21]. We were not able to link our data to individual-level health behavior (e.g. walking) or outcome (e.g. obesity) data, which could give us a better understanding on the effects of walkability in shaping health inequities. Our marker of gentrification is an unspecific one, as a change in education proportions may reflect both residential mobility phenomena (linked to gentrification) and changes in the non-moving population (linked to social mobility). Further research in Madrid with residential mobility data should explore the impact of these two phenomena on our inferences.

Our study supports the idea that low-income neighborhoods had a more-walkable urban form; however, neighborhood dynamics in terms of social composition (gentrified neighborhood) and in terms of neighborhood age (newest areas) did not follow the same pattern. These findings are key to understand how to address physical activity inequities within a city. If new neighborhoods in Madrid are built following a different socio-spatial distribution of walkability (more favorable for the wealthy, or with a loss of a walkability advantage for the poor), and the wealthy people are moving to the walkable neighborhoods [25], there is a need to balance with safeguards to preserve affordability and avoid the displacement of low-SES populations, keeping the “right to the city” with adequate housing reforms [44]. Therefore, continued attention needs to be paid to equity in urban policies to change the urban form to ensure changes do not have the unintended consequence of increased health inequities [45].

## Conclusions

In conclusion, our study shows that higher SES areas of Madrid had lower walkability compared with lower SES areas. However, neighborhood dynamics in terms of social (gentrification) an urban form (neighborhood age) modified this association; newest and gentrified neighborhoods had a flat curve between area-SES and walkability. A deeper understanding of the dynamic relationship between urban form and neighborhood composition would provide further insights into mobility and health behaviors and outcomes, and inform urban planning policy in European cities to preserve health equities.

## Additional files

**Additional file 1:** Additional information on the operationalization of the variables.

**Additional file 2. Table S2:** Census section sociodemographic and walkability indicators according to socioeconomic status (SES) tertiles (N = 2415).
