# Supplementary material for: Intersection of neighborhood dynamics and socioeconomic status in small-area walkability: the Heart Healthy Hoods project
Source: Int J Health Geogr. 2017 Jun 6;16:21. doi: 10.1186/s12942-017-0095-7 (PMC5461703; doi:10.1186/s12942-017-0095-7)
Supplement: Supplementary file 1 — Additional file 1: Additional information on the operationalization of the variables. [file 12942_2017_95_MOESM1_ESM.docx]

**Additional file 1: additional information on the operationalization of the variables**

**SES indicators**

The two education indicators were obtained from the Padron, a continuous census of the entire population used for administrative purposes. The three occupation indicators (part time jobs, temporal jobs, and manual occupation class) were obtained from the Social Security data; the denominator was the total number of workers. Property value was obtained from the Idealista Report, a yearly study of neighborhood-level sale prices of all housing sold through the biggest real state corporation in Spain (Idealista). Property value data from the IDEALISTA Report contains data for all houses listed for sale in their website on the first day of each year. The report contains data at the neighborhood level (n=128 each year). To translate this to the census section level, we obtained data from the IDEALISTA API (<http://developers.idealista.com/access-request>) on April 18^th^ 2016. We collected all housing units for sale on that day, including their price, size and geocoded location. We overlayed a census section polygon file and assigned each housing unit to a census section. With this, we constructed a measure of average property value per census section for 2016. We then used a weighted linear mixed model with property value at the census section as the dependent variable, and property value at the neighborhood level (from the IDEALISTA Report 2016 data) as a fixed and random coefficient (at the neighborhood level ,with an unstructured covariance structure), and the following fixed effects for each census section: % low education, % high education, % immigration from non-oecd countries, % people below age 25, % people above age 25, and a quadratic fixed term for each indicator. Each observation was weighted by the number of housing units on sale on each census section. We then predicted the property value in each census section in 2014 by replacing the data above with the respective data from 2014. To diagnose this imputation we correlated the predicted values for 2016 with the observed values in 2016, finding a pearson correlation coefficient of 0.93. Registered unemployment was obtained from the statistics of the Employment Service (SEPE); the denominator was, given the lack of a better measure for the active population at this level, the amount of people between 16 and 64 years of age in the neighborhood, obtained from the Padron. All data was downloaded from the statistics website of the City Government of Madrid.

**Neighborhood dynamics indicators**

Gentrification was obtained by ranking all census sections in 2005 and in 2014 in terms of % residents with high education (university education or above) and computing the change in rank from 2005 to 2014, where we defined a gentrified neighborhood as those in the top 95% percentile of rank change. Education data for this calculation was obtained from Padron. Median year of construction of all housing units in the census section was obtained from the Cadastre (*Catastro*, a universal tax registry of all housing units). We created three categories: up to 1985, from 1985 to 1997, from 1997 onwards.

**Walkability indicators**

Residential Density was operationalized as occupied dwellings by km^2^; occupied dwellings were obtained from housing census. Total residents’ data for the Population Density indicator (Residents/km^2^) was obtained from Padron. Retail and Service Destinations were obtained from the Retail Spaces Census at the Madrid Council Open Database, that includes data on economic activities of all occupied commercial spaces; from this dataset, we select the categories for Retail and Services (47, 53, 56, 85, 90,91, 92, 93, 96 categories). For street connectivity, we calculated a Kernel Density Estimation (KDE) in 3mx3m pixels of the density of street intersections, resulting on a pixel-based surface. KDE fits a mathematical surface (composed of pixels) with a normal distribution over each point based on (a) the value empirically collected for each point, and (b) the distance from each location in the surface to all points in the area within defined radius or bandwidth. Essentially, the value of each point is smoothed over the study area producing a density value that will be the highest at the location of every point, and decaying from there with distance using a defined bandwidth. We used de KDE integrated in ArcGis 10.1 software which employs the quadratic Kernel function of Silverman:

$$f\left( x \right)=\frac{1}{nh}+\sum_{i=1}^{n} K \left( \frac{x-x_{i}}{h} \right)$$

where K is the quadratic Kernel function defined by $K\left( x \right)=\frac{3}{4}(1-x^{2})$ , x ≤ 1, “x” is the point at which density is estimated, “xi” is the value of the variable in the case “i”, “n” is the number of cases and “h” is the bandwidth. The basic idea consists calculated for specific points, the averaged sum (hence the estimator involves summing over “n” and then divide by this value) of Kernels centered on the observations.
