# Supplementary material for: Intersection of neighborhood dynamics and socioeconomic status in small-area walkability: the Heart Healthy Hoods project
Source: Int J Health Geogr. 2017 Jun 6;16:21. doi: 10.1186/s12942-017-0095-7 (PMC5461703; doi:10.1186/s12942-017-0095-7)
Supplement: Supplementary file 2 — Additional file 2. Table S2: Census section sociodemographic and walkability indicators according to socioeconomic status (SES) tertiles (N = 2415). [file 12942_2017_95_MOESM2_ESM.docx]

**Additional file 2: Table S2.** Census section sociodemographic and walkability indicators according to socioeconomic status (SES) tertiles (N=2415)

| **Census section characteristic** | **Census section Socioeconomic Status** | | | |
| --- | --- | --- | --- | --- |
|  | **Tertile 1**  **(Low SES)** | **Tertile 2**  **(Mid SES)** | **Tertile 3**  **(High SES)** | **Overall** |
| N | 805 | 805 | 805 | 2415 |
| Population | 1186.0(967.0;1447.0) | 1211.0(948.0;1544.0) | 1269.0(1013.0;1628.0) | 1216.0(973.0;1544.0) |
| SES indicators |  |  |  |  |
| % of low Education | 70.2(64.9;75.9) | 49.7(41.1;57.2) | 24.9(19.2;31.5) | 49.5(30.0;66.1) |
| % of high Education | 11.5(8.1;15.0) | 27.0(21.0;34.6) | 52.4(45.7;59.5) | 27.0(14.2;46.9) |
| % of part time Jobs | 28.1(27.1;30.8) | 24.0(20.8;26.7) | 16.6(14.9;17.8) | 22.9(17.6;27.7) |
| % of temporal Jobs | 22.3(21.2;23.1) | 20.6(19.2;21.6) | 17.5(15.4;18.7) | 20.3(17.8;21.8) |
| % of manual Occupation Class | 42.0(39.5;47.0) | 35.9(30.5;37.4) | 23.0(19.0;24.9) | 35.9(24.9;40.0) |
| Housing Prices 10^3^ €/m^2^ | 1.6(1.4;1.8) | 2.3(2.1;2.6) | 3.6(3.3;4.2) | 2.3(1.8;3.3) |
| Unemployment Rate | 14.7(13.5;16.1) | 12.1(10.9;12.8) | 8.1(6.9;9.1) | 12.1(9.1;13.9) |
| SES Index | -0.9(-1.2;-0.7) | -0.1(-0.3;0.2) | 1.0(0.8;1.3) | -0.1(-0.7;0.8) |
| Walkability indicators |  |  |  |  |
| Residential Density (10^3^ res/km^2^) | 12.7(8.6;18.1) | 13.2(7.7;19.9) | 12.2(5.8;20.4) | 12.8(7.5;19.4) |
| Population Density (10^3^ pop/km^2^) | 33.8(23.3;45.4) | 30.8(19.6;45.0) | 29.6(15.3;46.5) | 31.8(19.4;45.5) |
| Retail Destinations Density (retail/km^2^) | 419.6(159.9;879.5) | 474.8(186.7;1259.5) | 669.6(189.5;1440.7) | 484.6(175.7;1169.7) |
| Street Connectivity (Kernel Density) | 0.2(0.1;0.3) | 0.2(0.1;0.3) | 0.1(0.1;0.2) | 0.2(0.1;0.3) |
| Walkability Index | 0.6(-1.1;2.1) | 0.4(-1.7;2.2) | 0.1(-2.3;2.1) | 0.4(-1.8;2.1) |
| Neighborhood Dynamics |  |  |  |  |
| % Gentrified in the last 10 years | 2.90% | 8.20% | 3.90% | 5.00% |
| % Median Year of Construction < 1985 | 87.80% | 80.20% | 80.40% | 82.80% |
| % Median Year of Construction 1985-1997 | 8.90% | 8.60% | 9.90% | 9.20% |
| % Median Year of Construction > 1997 | 3.20% | 11.20% | 9.70% | 8.00% |
